# Circulating fibroblast activation protein α is reduced in acute ischemic stroke

**DOI:** 10.3389/fcvm.2022.1064157

**Published:** 2022-12-07

**Authors:** Jan-Thorben Sieweke, Gerrit M. Grosse, Karin Weissenborn, Anselm A. Derda, Saskia Biber, Johann Bauersachs, Udo Bavendiek, Jochen Tillmanns

**Affiliations:** ^1^Department of Cardiology and Angiology, Hannover Medical School, Hannover, Germany; ^2^Department of Neurology, Hannover Medical School, Hannover, Germany

**Keywords:** fibroblast activation protein (FAP), ischemic stroke, echocardiography, atrial conduction time, septal PA-TDI

## Abstract

**Background:**

Fibroblast activation protein α (FAP), a membrane glycoprotein with dipeptidyl-peptidase and collagenase properties, is expressed in atherosclerotic plaques and remodeling of the extracellular matrix based on fibrosis. Fibrosis is a main contributor of atrial cardiomyopathies. In acute MI, circulating FAP is associated with outcome. Here, we investigated the correlation of circulating FAP to echocardiographic parameters of atrial remodeling and neurological impairment in acute ischemic stroke.

**Methods:**

Circulating FAP plasma concentrations were determined by ELISA in 47 patients with acute stroke and 22 control patients without stroke. Echocardiography was performed in all participants. Laboratory analysis, National Institutes of Health Stroke Scale (NIHSS) scoring and prolonged Holter-ECG-monitoring were performed in all stroke patients.

**Results:**

Patients with acute stroke had lower circulating FAP concentrations than the control cohort (92 ± 24 vs. 106 ± 22 ng/mL, *P* < 0.001). There was no difference between the circulating FAP concentration comparing stroke due to atrial fibrillation, embolic stroke of undetermined source (ESUS) or atherosclerotic origin. Septal atrial conduction time (sPA-TDI) and left atrial (LA) volume index to tissue Doppler velocity (LAVI/a‘) representing echocardiographic parameters of LA remodeling did not correlate with FAP concentrations (sPA-TDI: r = 0.123, *p* = 0.31; LAVI/a‘: r = 0.183, *p* = 0.132). Stroke severity as assessed by NIHSS inversely correlated with circulating FAP (r = −0.318, *p* = 0.04). FAP concentration had a fair accuracy for identifying stroke in the receiver operating characteristic (ROC) analysis (AUC = 0.710, 95% CI: 0.577–0.843). A FAP concentration of 101 ng/mL discriminated between presence and absence of stroke with a sensitivity of 72% and a specificity of 77%. Lower circulating FAP concentration was associated with cardio-cerebro-vascular events within 12 months after admission.

**Conclusions:**

Our study is the first to associate FAP with echocardiographic parameters of LA-remodeling and function. FAP did not correlate with sPA-TDI and LAVI/a‘. However, FAP was associated with stroke, neurological impairment, and cardio-cerebral events within 12 months. Therefore, FAP might enable individualized risk stratification in ischemic stroke.

## Introduction

In western countries stroke is one of the most common reasons for death and disability (1). There is a substantial need for early diagnosis of stroke and risk stratification of neurological impairment.

Atrial fibrillation (AF) is a major etiology of stroke (2). Atrial cardiomyopathy, characterized by remodeling and fibrosis, contributes to the onset of AF (3, 4). Recently, we identified the association between circulating microRNA 21 and NT-proBNP with AF (5–7). Furthermore, these biomarkers correlated with septal atrial conduction time (sPA-TDI) and left atrial volume indexed to tissue Doppler velocity (LAVI/a‘), echocardiographic parameters of left atrial function and remodeling predicting AF (5, 8). Biomarkers might be useful to assist in diagnosing stroke and estimating individual risk of neurological impairment after stroke. However, at present no biomarker is known to aid in the individual diagnosis of stroke, therapeutic stratification and hospital management (9).

The dipeptidyl-peptidase fibroblast activation protein α (FAP) is a serine protease membrane glycoprotein with dipeptidyl-peptidase and collagenase properties, expressed by activated fibroblasts after myocardial infarction (MI) in animals and humans (10, 11). It is also expressed in atherosclerotic plaques (12). In acute MI, circulating FAP is associated with the risk of death and compared to healthy blood donors, circulating FAP concentrations were reduced in patients with acute coronary syndrome (13). Circulating FAP has gained attraction as a marker of the progression of atherosclerotic plaques and remodeling of the extracellular matrix based on fibrosis (14). Previously published work showed that FAP concentration changes over time after stroke (14, 15).

Hence, FAP seems to be a potential candidate for risk stratification in patients with cardio-vascular disease. Therefore, the present study investigated the hypothesis that circulating FAP concentration correlates with echocardiographic parameters representing left atrial remodeling and discriminates patients with ischemic stroke against those without stroke. Furthermore, we hypothesized that circulating FAP concentrations correlate with stroke severity and the occurrence of recurrent cardio-cerebrovascular events in the long-term course.

## Methods

### Study design and participants

The present study is a prospective, semi-blinded, single-center controlled study. The local ethics committee of Hannover Medical School approved this study (application number: 3316-2016). The study complies with the Declaration of Helsinki. All participants gave written informed consent. This study is a sub-study of a recently published work describing the prediction of AF by echocardiographic parameters in patients (>50 years of age) after embolic stroke of undetermined source (ESUS) and control cohorts. Detailed information on in-/exclusion criteria and clinical work-flow have published previously (8).

The primary objective of this analysis was to investigate the correlation of circulating FAP concentrations with echocardiographic of left atrial remodeling and function (sPA-TDI, LAVI/a‘). The secondary objective was (I) to clarify the discrimination ability of cerebral infarction by circulating FAP and (II) the correlation of FAP with risk factors for cardiovascular events and with stroke severity.

Subsequently, included participants were categorized into cohorts and groups:

1) Control cohort: participants without stroke (*n* = 22).
2) Stroke-cohort: patients with stroke (*n* = 47). To clarify the secondary objective of this analysis, the cohort of stroke patients was divided according to their underlying etiology into ESUS (*n* = 20), stroke due to AF (*n* = 10), and cerebral infarction of atherosclerotic origin (*n* = 17). Before a scheduled Holter ECG-Monitoring, patients classification to stroke groups was performed including brain imaging, continuous ECG monitoring with a central monitoring unit (at least 24 h), 12-channel surface ECG recordings, echocardiography and additional examinations if indicated. Across cohorts, a total of 20 patients with AF were included in the analysis (30% of patients).

After echocardiographic examination, blood samples were collected. In patients with stroke, blood samples were taken between the 3^rd^ and 7^th^ day after stroke-onset.

According to the American Society of Echocardiography guidelines a dedicated and detailed transthoracic echocardiography (16) and a 12-lead electrocardiogram (ECG) was performed in all participants. Echocardiographic parameters of LA-function and –remodeling were determined as recently described (8). In patients after stroke, a Holter-ECG-monitoring was scheduled for 72 h. Two independent and blinded professionals applying current guidelines on AF analyzed all ECG recordings (17).

### 12-months post discharge follow-up

Patient status was followed-up over 365 days after enrollment of all patients (*n* = 47). Cardio-cerebro-vascular events were recorded according to a case-report form as described previously (18).

### Control cohort

We obtained EDTA plasma samples after transthoracic echocardiography from 22 volunteer blood donors presenting with sinus rhythm, who were admitted to the cardiology ward at Hannover Medical School between August 2016 and May 2017. This control cohort included patients with known paroxysmal AF hospitalized for catheter ablation of AF [*n* = 10 (45.5%)]. Furthermore, patients with suspected chronic heart failure due to dyspnea [*n* = 6, (27%)], patients with suspected progression of CAD [*n* = 3, (14%)], patients with arterial hypertension [*n* = 2, (1%)], and a patient after suffering from syncope (0.5%) were included in the control cohort.

Individuals with acute myocardial infarction, history of myocardial infarction, acute stroke, chronic drug or alcohol abuse, malignant disease, recent immunizations, recent major or minor surgical treatments, age under 50 years, AF during echocardiographic examination, severe mitral valve stenosis or regurgitation, history of aortic or mitral valve replacement, ablation of supraventricular tachycardia and participants, who could not provide informed consent were excluded as controls from the analysis.

All subjects of the control cohort gave informed consent to donation and blood sampling for research purposes. All participants of the control cohort underwent a 12-lead electrocardiogram and transthoracic echocardiogram.

### Stroke cohort

We obtained EDTA plasma samples from 47 patients with stroke and minor neurologic deficits [median National Institutes of Health Stroke Scale (NIHSS) of 2 (IQR 2-5), who were treated at our certified Stroke Unit at Hannover Medical School between August 2016 and May 2017. Plasma samples were obtained between the 3^rd^ and 7^th^ day after stroke, and stored at−80°C. Patients presenting in sinus rhythm and with acute focal neurological symptoms with accompanying evidence of cerebral infarction on magnetic resonance imaging or cranial computer tomography were included in the stroke cohort. Stroke patients with vasculitis, endocarditis, and dissection of the brain supplying arteries as etiology were excluded. Further exclusion criteria were defined as follows: age under 50 years, acute or chronic history of myocardial infarction, severe stenosis or regurgitation of the mitral valve, history of aortic or mitral valve replacement, ablation of supraventricular tachycardia, hemorrhagic stroke, AF during echocardiographic examination and patients, who could not provide informed consent and received thrombolytic treatment. At admission, Stroke physicians determined neurological impairment according to the National Institutes of Health Stroke Scale (NIHSS). Patients who consented to a blood draw for study purposes and had it deposited by the Department of Cardiology were included in the study.

### FAP-ELISA and routine laboratory analysis

Measurement of FAP was performed in EDTA plasma using a commercial ELISA (R&D Systems DY3715) as described previously (13). Each sample was analyzed in duplicate, and pooled control plasma samples (stored in aliquots at−80°C) were analyzed with every single run. Pre-analytic characteristics of FAP and technical characteristics of the FAP-ELISA were described previously (13).

Routine laboratory analysis of C-reactive protein (CRP) was performed in blood samples taken on the same day as FAP plasma samples. Creatine kinase (CK) was analyzed on admission. These two biomarkers were analyzed at the Department of Clinical Chemistry at Hannover Medical School in clinical routine.

The operators performing the ELISA and the routine laboratory analysis were blinded regarding patient characteristics of all samples analyzed and were not involved in data analysis. The authors were not involved in sample handling or laboratory analyses.

### Statistical analysis

Statistical analysis and graphical presentation were performed using SPSS Statistics 25 (IBM SPSS Statistics 25, Chicago, IL, USA) and GraphPad Prism 7.04 (Graph Pad Software, San Diego, CA, USA). Categorical variables are presented as *n* (%), normally distributed continuous variables as mean ± standard deviation (SD), and non-normally distributed continuous variables as median and interquartile ranges (IQR). Normality and variance homogeneity were checked by Shapiro-Wilk and D‘Agostino Pearson test. The groups were compared using Student's *t*-test for Gaussian distributed data and the Mann-Whitney U test in non-normally distributed data. ANOVA was performed followed by Bonferroni test or Dunn‘s test for multiple comparisons, respectively. Categorical variables were evaluated by Chi-Square test. Correlations between variables and FAP were tested with bivariate Pearson correlation or Spearman correlation, as appropriate.

The discriminative ability of FAP was assessed by the area under the receiver operating characteristic (ROC) curve. Cut-off values for prediction were defined as the cut-off point having the highest Youden index (Yi = sensitivity + specificity - 1). Sensitivity, specificity, positive and negative predictive value, accuracy for determining cut-offs were calculated. Cumulative incidence of cerebro-vascular events within 12-months follow-up was estimated by Kaplan-Meier method and the stroke cohort dichotomized by the median of FAP were compared by the log-rank test. A two-sided *P*-value of <0.05 was considered statistically significant.

## Results

### FAP concentrations in control cohort

**Figure 1:**
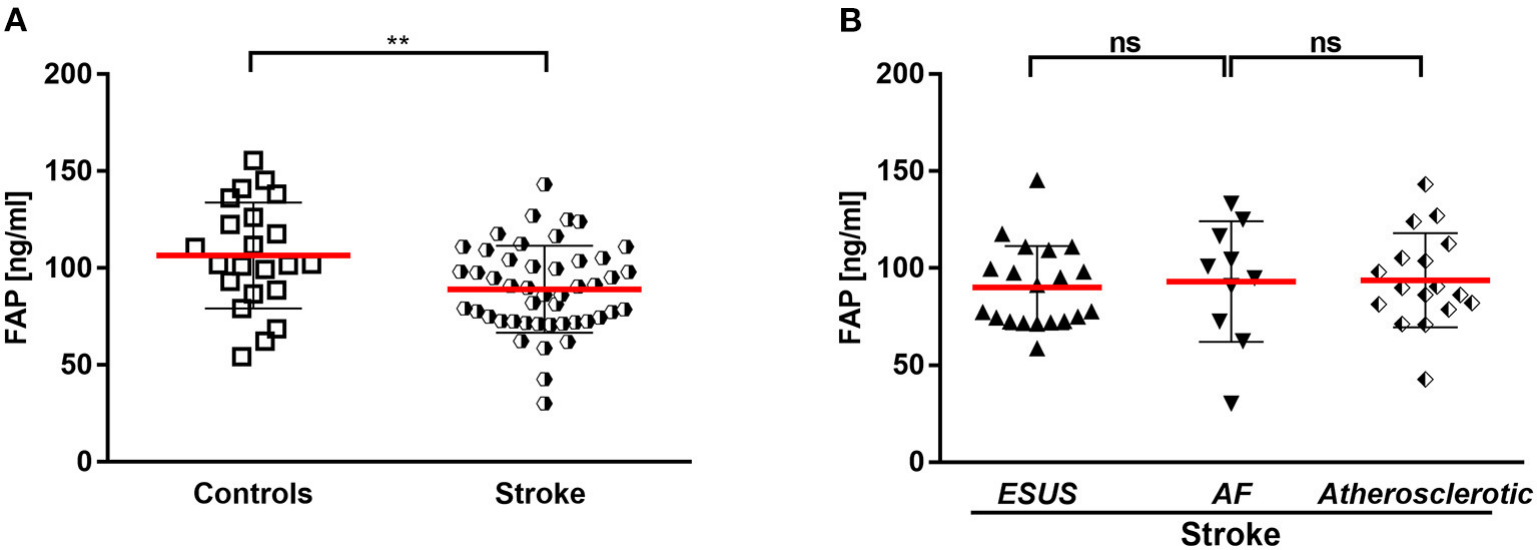
**(A)** Circulating FAP in stroke and controls. **(B)** Circulating FAP in stroke entities. AF, atrial fibrillation; ESUS, embolic stroke of undetermined source. **p < 0.01.

A collection of 22 EDTA plasma samples were taken from a control cohort [73% men, median age of 69 (IQR 57–77 years)] and 27% women, median age of 68 (IQR 58–78 years), and assayed using the FAP ELISA. Control cohort samples had a mean FAP concentration of 106 ± 22 ng/mL (Figure 1A). FAP concentrations in men [median 113 (IQR 97–137 ng/mL)] and women [median 102 (IQR 91–110 ng/mL)] did not differ significantly (*P* = 0.34). Baseline characteristics of the control cohort are displayed in Table 1.

**Table 1:** Patient characteristics.

**Parameter**	**Control (*n* = 22)**	**Stroke** **(*n* = 47)**	***P***	**Stroke**		
				**ESUS (*n* = 20)**	**AF (*n* = 10)**	**Vascular (*n* = 17)**
Age [years]	69 [57–77]	71 [60–79]	ns	63 [51–76]	78 [72–81]	72 [64–80]
Male	16 (73%)	31 (66%)		15 (75%)	5 (50%)	11 (64%)
BMI [kg/m^2^]	27 ± 2.7	27 ± 5.4	ns	25.8 ± 3.1	25.9 ± 5.4	29 ± 7
**Pre-existing conditions**						
Hypertension	13 (59%)	35 (75%)	ns	13 (65%)	9 (90%)	13 (77%)
Diabetes	2 (9%)	14 (30%)	ns	3 (15%)	4 (40%)	7 (41%)
Smoking	6 (27%)	10 (21%)	ns	5 (25%)	1 (10%)	4 (24%)
Stroke	4 (18%)	13 (28%)	ns	2(10%)	2 (20%)	9 (53%)*
CKI	0	6 (13%)	ns	2 (10%)	1 (10%)	3 (18%)
Heart failure	1 (5%)	1 (2%)	ns	0	0	1 (6%)
CAD	5 (23%)	10 (21%)	ns	2 (10%)	3 (30%)	5 (29.4%)
PAD	3 (14%)	4 (9%)	ns	1 (5%)	0	3 (18%)
NIHSS	n.a.	2 [1–5]		2 [1–3]	1 [1–5]	3 [2–5]
CK [U/L]	96 [56–151]	91 [58–149]	ns	102 [63–140]	79 [54–164]	85 [53–147]
CRP	1.9 [1–4.9]	2.3 [1.2–6.2]	ns	2.1 [1.2–6.7]	2.4 [1–9.1]	3 [1.5–4.9]
LVEF [%]	60.4 ± 6.4	59.5 ± 6.6	ns	62 ± 4.1	59.3 ± 5.8	58.2 ± 5.4
LAVI [ml/m^2^]	36.6 ± 10.7	34.5 ± 12.1	ns	31.2 ± 8.7^#^	47.9 ± 15	29.4 ± 7.2^#^
PA-TDI septal	105.3 ± 27.9	101.5 ± 21.8	ns	88.3 ± 12.1^###^	137 ± 5.5	96.1 ± 12^###^
PA-TDI lateral	117 ± 28.3	111.4 ± 20.5	ns	100.6 ± 14.7^###^	143.4 ± 7.6	105 ± 9.8^###^
LAVI/a‘	3.6 [2.9–4.3]	3.2 [2.5–4.1]	ns	2.9 [1.8–3.7]^##^	7.2 [3.6–8.7]	3 [2.5–3.5]^##^

AF, atrial fibrillation; BMI, body mass index; CAD, coronary artery disease; CK, creatinine kinase; CKI, chronic kidney disease; CRP, C-reactive protein; ESUS, embolic stroke of undetermined source; LAVI, left atrial volume index; LV, left ventricular; LVEF, left ventricular ejection fraction; NIHSS, National Institutes of Health Stroke Scale; PAD, peripheral artery disease. Variables are expressed as mean ± SD, median [IQR] or n (% of total number). *p < 0.05 vs. ESUS, ^#^p < 0.05 vs. AF, ^*##*^p < 0.01 vs. AF, ^*###*^p < 0.001 vs. AF.

### FAP concentrations in stroke patients

FAP concentrations were measured in EDTA plasma samples from 47 patients admitted with acute ischemic stroke, comprising 31 (66%) men with a median age of 70 (IQR 58–78) years and 16 (34%) women with a median age of 74 (IQR 64–80) years.

Stroke patients [men and women, median age 70 years (IQR 60–79)] did not differ significantly in age in comparison to the control cohort [men and women, median age 69 years (IQR: 57–77)] (*p* = 0.56). The sex distribution was not significantly different between the patients and the control cohort (p: 0.32). By echocardiography, systolic LV function was not different between cohorts (Table 1). Of note, CRP levels were very low [stroke patients: 2.3 mg/L (1.2–6.2); controls: 1.4 mg/L (0.5–3.7)] and not different in both cohorts (*p* = 0.2). Baseline characteristics of the stroke cohort are presented in Table 1.

Stroke patients had a mean FAP concentration of 92 ± 24 ng/mL after the stroke event (median: 7 days; IQR 4–7 days), which was significantly lower compared with the control cohort (*P* < 0.001) (Figure 1A).

In the stroke cohort (*n* = 47), etiology was of atherosclerotic origin in 36%, AF in 21 and 43% embolic stroke of undetermined source until discharge (ESUS). There was no difference between the FAP concentrations in the stroke subcohorts ESUS, AF and vascular cause (Figure 1B).

FAP concentrations in stroke were not different in men (mean 91 ± 21 ng/mL) and in women (mean 88 ± 27 ng/mL) (*p* = 0.64).

### FAP and markers of cardiovascular disease

**Figure 2:**
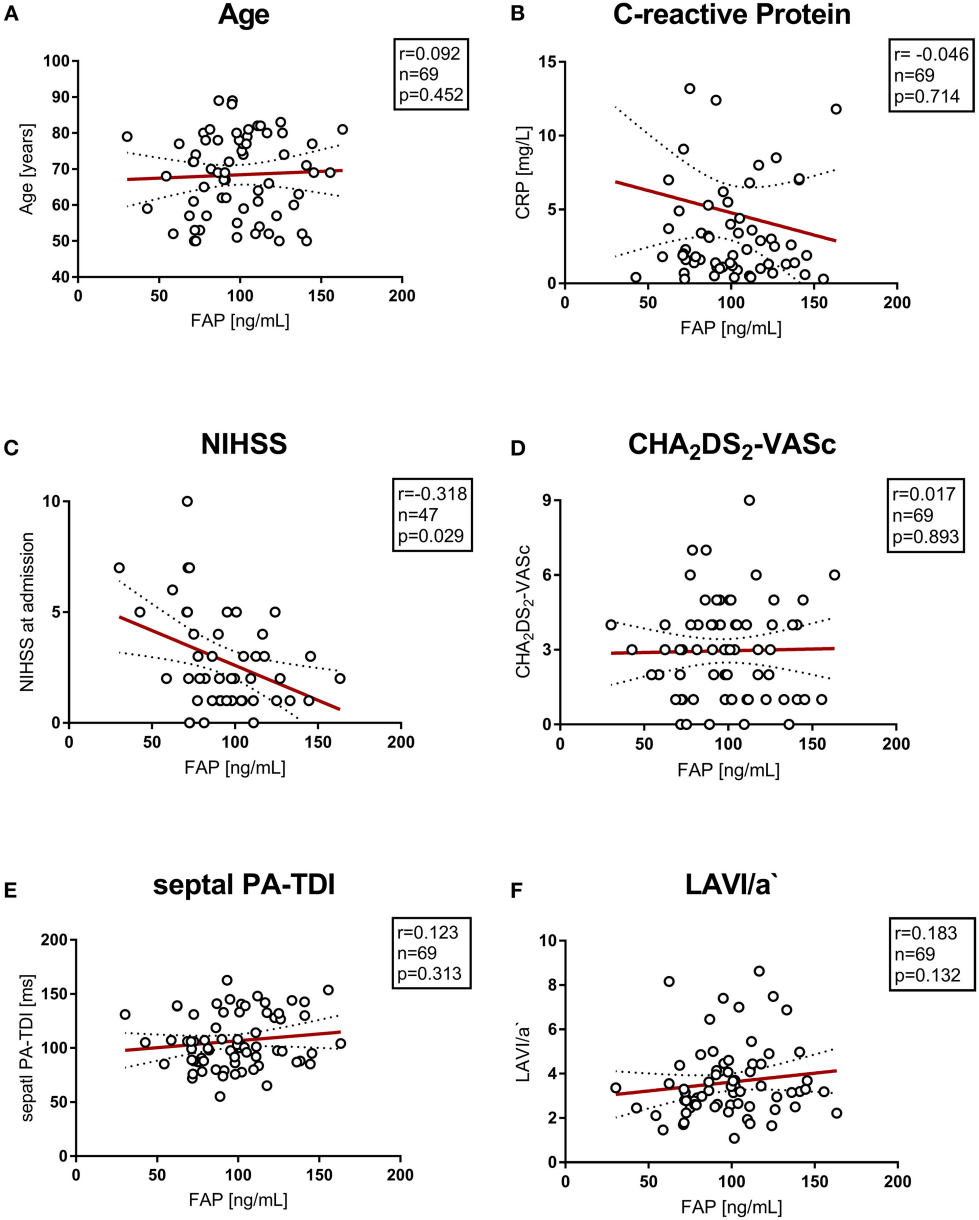
**(A–F)** Correlation of vascular risk scores, biomarkers and echocardiographic parameters with circulating FAP values. NIHSS, National Institutes of Health Stroke Scale; PA-TDI, total atrial conduction time.

There was no correlation of circulating FAP concentration with levels of C-reactive protein and creatinine kinase or impaired renal function. Echocardiographic parameters associated with AF and left atrial remodeling and function (septal PA-TDI and LAVI/a) did not correlate with FAP. Also, the parameter of diastolic function E/E' was not correlated with circulating FAP concentrations after stroke. Furthermore, FAP concentration did not correlate with cardio-vascular disease and cardio-embolic risk represented by CHA_2_DS_2_-VASc score and NT-proBNP. Of note, FAP concentration inversely correlated with stroke severity assessed by NIHSS (r = −0.318, *p* = 0.03). Correlations of FAP concentrations with markers of cardiovascular disease are presented in Figure 2, Supplementary Figure 1.

### FAP as discriminator for the presence of stroke

**Figure 3:**
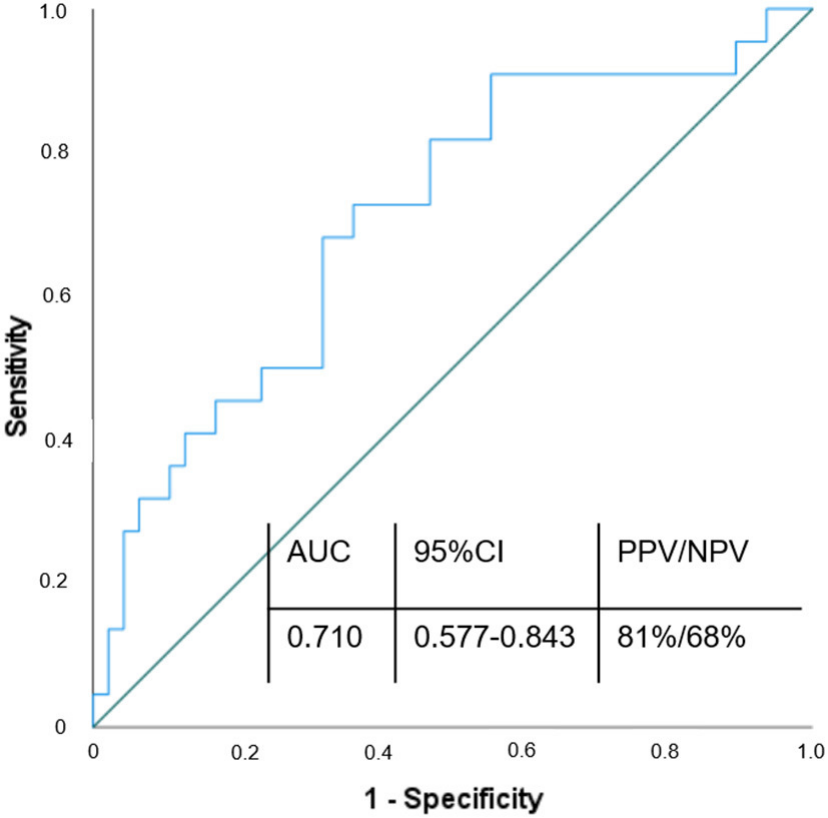
ROC curve of FAP in diagnosis of ischemic stroke. Variable of state: w/o stroke; AUC, Area under the curve; CI, Confidence interval; PPV, Positive predictive value; NPV, Negative predictive value.

To evaluate the ability of circulating FAP concentrations to discriminate patients with stroke from patients without stroke, we performed a ROC analysis. ROC showed a fair discriminative ability of FAP for the presence of stroke (AUC = 0.710, 95% CI 0.577–0.843). Subsequent analysis of Youdens index determined discriminators of FAP for the presence of stroke as follows: 101 ng/ml [sensitivity 72% (95% CI 67.36–84.38%); specificity 77% (95% CI 59.86–89.58%); Positive Predictive Value 81% (95% CI 69.28–88.9%); Negative Predictive Value 68% (95% CI 55.84–77.33%)]. ROC-analysis is provided in Figure 3.

## Twelve months follow-up

**Figure 4:**
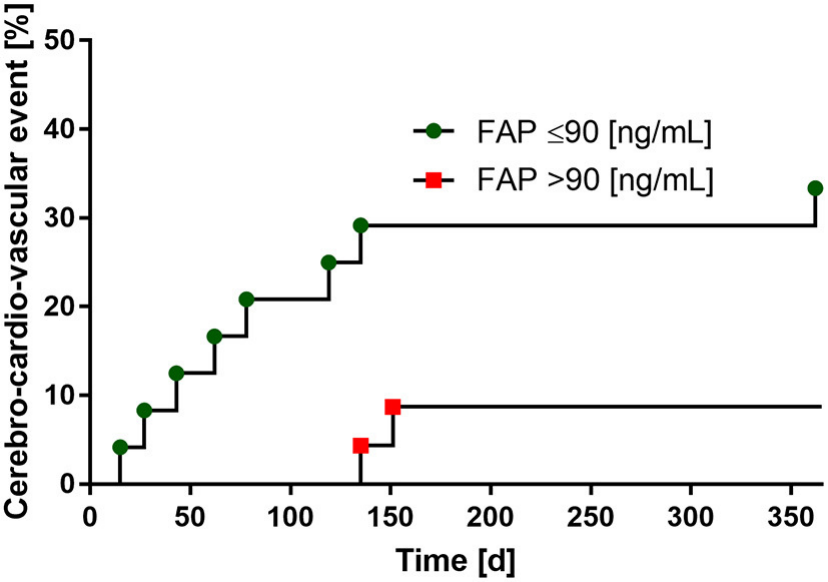
Low FAP-levels are associated with the risk of recurrent cerebro-cardio-vascular events within 1 year follow-up. FAP values dichotomized by the median (90 ng/mL).

In the 12-month follow-up after index event of patients with stroke, a total of 10 cardio-cerebro-vascular complications with subsequent hospitalization occurred. Of these, seven were recurrent strokes and three cases were myocardial infarctions. When dichotomized by the median of FAP values (median: 90 ng/mL), we found an association between low FAP concentrations and the consequence of a recurrent cardio-cerebro-vascular event within 365 days after stroke, which did not reach statistical significance (*p* = 0.061). Results of the 12-months follow-up are provided in Figure 4. No patient was lost to follow-up.

## Discussion

Risk stratification in acute stroke have decisive influence on the treatment and secondary prevention of stroke. Circulating biomarkers associated with thrombo-embolic events may represent diagnostic targets and allow risk stratification in stroke. FAP is a membrane-associated serine protease expressed by activated fibroblast and is associated with myocardial damage and prognosis in myocardial infarction (13, 19).

We evaluated the concentration of FAP in plasma of a control cohort consisting of volunteer blood donors and stable patients admitted to the cardiology ward at Hannover Medical School. FAP concentrations in the control cohort had a mean of 106 ± 22 ng/ml ng/ml. These results correspond well to FAP concentrations previously found in apparently healthy blood donors (13) after applying in- and exclusion criteria of the current analysis as provided in Supplementary Figure 2.

Circulating FAP concentrations in stroke patients were 19% lower than in the control cohort. Age was not different between stroke patients and control cohort, and there was no correlation between FAP concentrations and age. These results are similar to a previous study, showing that patients with acute coronary syndrome had lower FAP concentration than controls. There was no correlation of FAP concentration with age in this large population of healthy blood donors and patients admitted with acute coronary syndrome (13). Similarly, no association between FAP concentrations and age was found in patients with stroke, coronary heart disease and control individuals in another study by Uitte de Willige et al. (14).

In patients with stroke, blood samples were taken between the 3^rd^ and 7^th^ day after stroke-onset to avoid interactions within the acute phase, because it has already been shown that the expression of FAP is induced by macrophage-derived TNFα which is a mediator of post-stroke inflammation (12). Importantly, while reduced FAP enzyme activity in the setting of stroke has been shown (15), to the best of our knowledge, our data show for the first time reduced FAP concentrations after stroke. This corresponds well with results from Baerts et al. (15) reporting a decrease in FAP enzymatic activity in the first week after stroke. Furthermore, FAP concentrations were shown to increase within months after a thrombo-embolic event suggesting decreased concentrations in the acute event (14).

Until today, the origin of circulating FAP is unknown. Previous studies suggest that circulating FAP does not reflect expression levels in organ tissues (13, 20). However, FAP can be used as a target structure by radiolabeled FAP-ligands for diagnostics by positron emission tomography in malignant tumors and myocardial injury (11, 21). The reason for decreased FAP concentrations after stroke in our study remains unknown. In patients with acute ST-elevation myocardial infarction FAP concentrations decreased from baseline levels within 5 days after the event and higher reductions of FAP concentrations were associated with larger myocardial infarct size (13, 19). Our finding, that FAP concentrations are associated with neurological impairment, is supported by a recently published paper that showed that larger infarcts were associated with a greater decrease in FAP activity (15).

Uitte de Willige et al. showed that circulation FAP concentrations were correlated with time after a thrombo-embolic event in coronary heart disease patients (14). We argue that reduced FAP concentrations after stroke might be associated with the thrombo-embolic event: Correlation of circulating FAP with cleavage of alpha 2 antiplasmin has been demonstrated (14), indicating a possible role in fibrinolysis of acute thrombus burden. FAP belongs to the dipeptidyl peptidase (DPP) four enzyme family, which have hydrolytic properties. FAP has dipeptidyl peptidase and endopeptidase activities, its substrates are largely unknown (22). Preclinical models targeting FAP show that FAP knockout is not associated with disease (23, 24) Circulating FAP cleaves alpha2-antiplasmin, which thereby becomes a potent inhibitor of plasmin, leading to a reduction in fibrinolysis (22). This fact supports our findings by higher NIHSS scores in patients with lower FAP concentrations. Furthermore, lower FAP concentrations in stroke patients were associated with a cardio-cerebral event within 12-months after admission. However, in our study we did not include patients with major neurological impairment and further studies will be necessary to evaluate a possible association between neurological impairment after stroke and FAP concentrations.

We further analyzed the correlation of various markers of cardiovascular disease with FAP concentrations in control and stroke cohorts. Neither inflammation, as evidenced by CRP-levels, nor overall stroke risk (CHA_2_DS_2_ VASc score), biomarkers of cardiovascular risk (NT-proBNP, CK) and echocardiographic parameters of atrial remodeling (septal PA TDI, LAVI/a) correlated with FAP concentration in our cohorts.

Total atrial conduction time (septal PA-TDI) is associated with fibrosis of the left atrium (25) and is associated with and predicting AF in patients suffering stroke presenting in sinus rhythm (8). Whereas, LAVI/a‘ is a marker of atrial remodeling, which is increased in AF patients (8, 25, 26). In the present analysis, FAP concentration and parameters of atrial function and remodeling did not correlate with each other, supporting the fact that FAP is a biomarker independent of the presence of atrial cardiomyopathies.

Inclusion criteria for stroke patients in this study were an acute focal neurological symptom with accompanying evidence of cerebral infarction in diffusion weighted (DWI) magnetic resonance imaging (MRI) or in cranial computer tomography (CT) with CT-angiography. So far, no biomarkers are available to aid diagnosis in clinical routine (9). More importantly, markers that could even predict the presence of strokes are not available yet. However, recent experimental analyses of glial fibrillary acidic protein and neurofilament light have also yielded hopeful data (27, 28).

Well known risk factors associated with stroke are hypertension, smoking, diabetes, and history of stroke (29). In our study, the number of patients with typical risk factors (hypertension, diabetes, stroke, chronic kidney injury) was higher compared to the control cohort. Of note, FAP was the only parameter associated with the presence of stroke in our study. There was no difference between the FAP concentrations in the stroke subcohorts with ESUS and stroke of AF and vascular origin.

For the first time, we established a ROC curve demonstrating the diagnostic value of FAP in discriminating between patients with and without stroke, yielding an AUC of 0.710. Using a FAP concentration cutoff of >101 ng/ml, we calculated a sensitivity of 72%, specificity of 77%, positive predictive value of 81 % and negative predictive value of 68% to establish the diagnosis of stroke. Additionally, we show for the first time that FAP inversely correlates with stroke severity and that low FAP concentration (below 90 ng/mL) seem to be associated with higher risk of stroke or MI within 1 year after the acute event.

## Study limitations

In this study, possible confounders must be taken into account. Due to the study design patients were taking medication at inclusion (provided in Supplementary Table 1). However, to the best of our knowledge, FAP concentrations are not effected by medication for diabetes or lipid disorders. No blood pressure trajectories were collected at defined time points. Baerts et al. showed a correlation of systolic and diastolic blood pressures with FAP activity (15). Additionally, a certain proportion of patients with stroke presented without precise information on the time of onset of the first neurological deficits. However, the duration between onset of neurological deficit and arrival in the emergency department was a median of 5 [3–15] h, so the influence of possible latency on FAP concentration may be small, especially because in the presence of low neurological deficit, FAP activity is comparable between days 3 and 7 (15). Sex differences of the circulating FAP concentration was described in a previous study. However, this difference could also not be demonstrated in a collective of patients with acute coronary syndrome (13, 14).

In our study, the number of stroke patients was rather small, and patients had minor neurological deficits. Therefore, this study should be repeated in a larger cohort, including measurements of circulating FAP on admission as well as the measurement of brain damage. It remains unclear whether FAP concentrations are already reduced before or just after stroke, indicating the need for further studies. Using FAP concentrations, it might be possible to discriminate strokes from stroke mimics and functional outcomes. We did not see different concentrations of FAP concerning stroke etiology. Therefore, it seems not possible to elucidate the etiology of stroke using this biomarker.

## Summary/conclusions

The present study reports decreased circulating concentrations of FAP in patients admitted with acute ischemic stroke. In contrast to established cardiovascular risk factors, low FAP concentrations were the only parameter associated with stroke in the first week after the event, and were associated with a higher incidence of stroke or MI events in the long-term course. FAP did not correlate with echocardiographic parameters of left atrial remodeling. Further studies are needed to evaluate the potential role of FAP as a novel biomarker in stroke and to establish whether low circulating FAP concentrations are a result of or can even serve as a predictor of future strokes or MI.

## Data availability statement

The original contributions presented in the study are included in the article/Supplementary material, further inquiries can be directed to the corresponding author.

## Ethics statement

The studies involving human participants were reviewed and approved by Local Ethics Committee of Hannover Medical School approved this study (Application Number: 3316-2016). The patients/participants provided their written informed consent to participate in this study.

## Author contributions

J-TS and JT edited the final draft and conceptualization of the analysis. J-TS, GMG, AAD, JB, UB, and JT wrote the manuscript. J-TS, GMG, KW, AAD, SB, JB, UB, and JT revised the manuscript critically. J-TS, JB, UB, and JT accurately approved the manuscript. All authors acquired an analyzed data and agree to be accountable for all aspects of the work ensuring that questions related to the accuracy or integrity of any part of the work are appropriately investigated and resolved, contributed to the article, and approved the submitted version.

## Supplementary material

Supplementary Figure 1 Correlation of vascular biomarkers with circulating FAP values.

Supplementary Figure 2 Comparison of circulating FAP concentrations in control cohorts. After applying the inclusion and exclusion criteria to the control cohort of Tillmanns et al. (13) comparable circulating FAP concentrations were displayed in both control cohorts (*p* = 0.86).

Supplementary Table 1 Medication at inclusion. ^1^blood pressure lowering drugs included: angiotensin-I antagonists, ACE-inhibitors, ß-blockers, mineralocorticoid receptor antagonists, calcium antagonists. ^2^Antikoagulation included: Apixaban, Rivaroxaban, Edoxaban. ^3^Antiplatelet therapy included: Acetylsalicyl acid and Clopidogrel.
