# Supplementary figures and images for: Circulating fibroblast activation protein α is reduced in acute ischemic stroke
Source: Front Cardiovasc Med. 2022 Dec 7;9:1064157. doi: 10.3389/fcvm.2022.1064157 (PMC9768027; doi:10.3389/fcvm.2022.1064157)

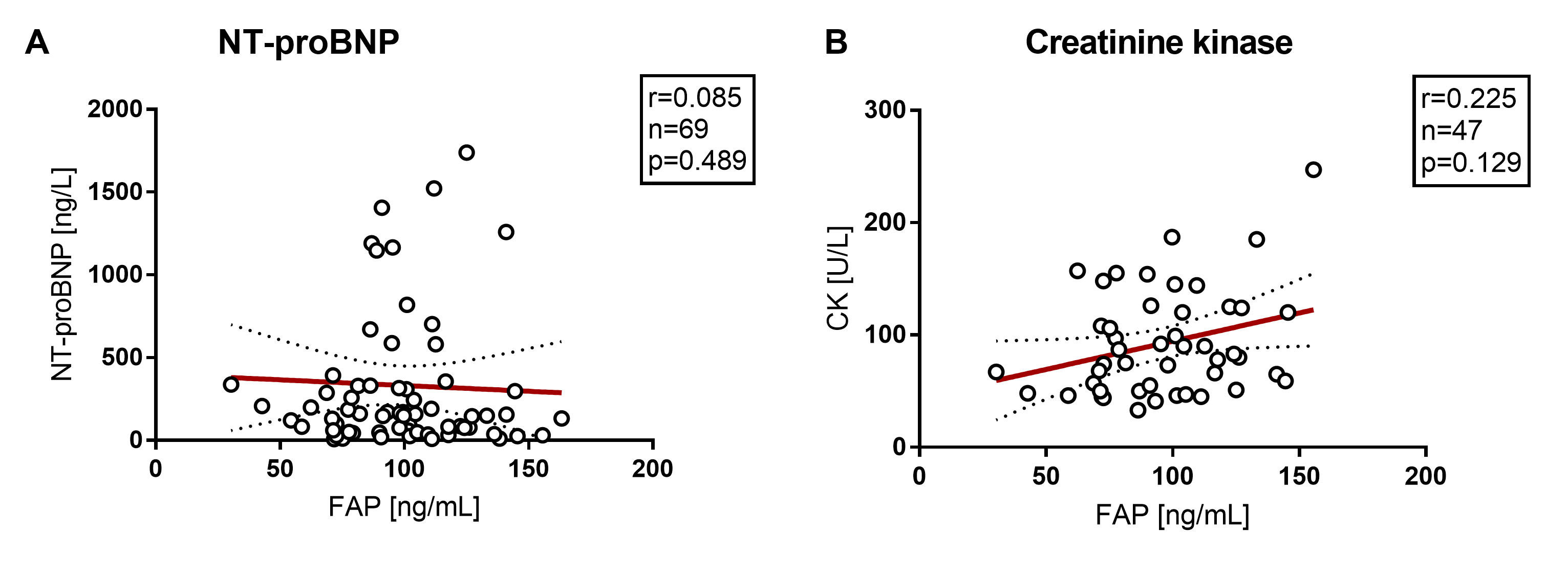

Supplement: Supplementary Figure 1 — Correlation of vascular biomarkers with circulating FAP values. [file Image_1.TIF]

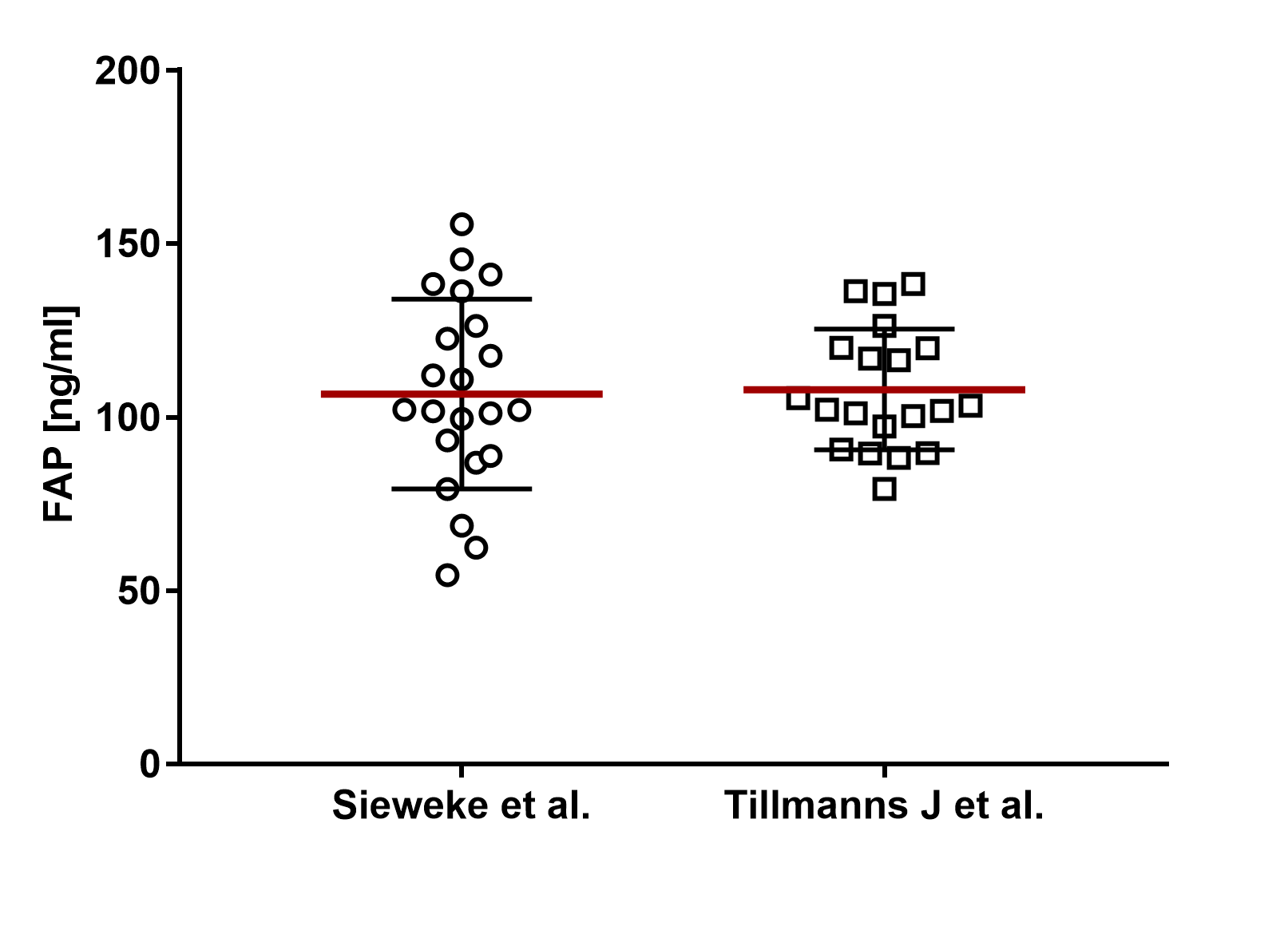

Supplement: Supplementary Figure 2 — Comparison of circulating FAP concentrations in control cohorts. After applying the inclusion and exclusion criteria to the control cohort of Tillmanns et al. (13) comparable circulating FAP concentrations were displayed in both control cohorts (p = 0.86). [file Image_2.TIF]
