# Supplementary material for: Circulating fibroblast activation protein α is reduced in acute ischemic stroke
Source: Front Cardiovasc Med. 2022 Dec 7;9:1064157. doi: 10.3389/fcvm.2022.1064157 (PMC9768027; doi:10.3389/fcvm.2022.1064157)
Supplement: Supplementary Table 1 — Medication at inclusion. 1blood pressure lowering drugs included: angiotensin-I antagonists, ACE-inhibitors, ß-blockers, mineralocorticoid receptor antagonists, calcium antagonists. 2Antikoagulation included: Apixaban, Rivaroxaban, Edoxaban. 3Antiplatelet therapy included: Acetylsalicyl acid and Clopidogrel. [file Table_1.docx]

| **Medication** | **Control**  (n=22) | **Stroke**  (n=47) | **p** |
| --- | --- | --- | --- |
| Diabetes | 2 (9%) | 12 (26%) | ns |
| Statins | 12 (55%) | 20 (43%) | ns |
| Blood pressure lowering drugs ^1^ | 12 (55%) | 30 (64%) | ns |
| Anticoagulation^2^ | 10 (46%) | 6 (13%) | <0.01 |
| Antiplatelet therapy ^3^ | 6 (27%) | 19 (40%) | ns |

**Supplementary Table 1:** Medication at inclusion

^1^blood pressure lowering drugs included: angiotensin-I antagonists, ACE-inhibitors, ß-blockers, mineralocorticoid receptor antagonists, calcium antagonists. ^2^Anticoagulation included: Apixaban, Rivaroxaban, Edoxaban.

^3^Antiplatelet therapy included: Acetylsalicyl acid and Clopidogrel.
